# Supplementary material for: FTO-mediated cytoplasmic m6Am demethylation adjusts stem-like properties in colorectal cancer cell
Source: Nat Commun. 2021 Mar 19;12:1716. doi: 10.1038/s41467-021-21758-4 (PMC7979729; doi:10.1038/s41467-021-21758-4)
Supplement: Supplementary file 1 — Supplementary Information [file 41467_2021_21758_MOESM1_ESM.pdf]

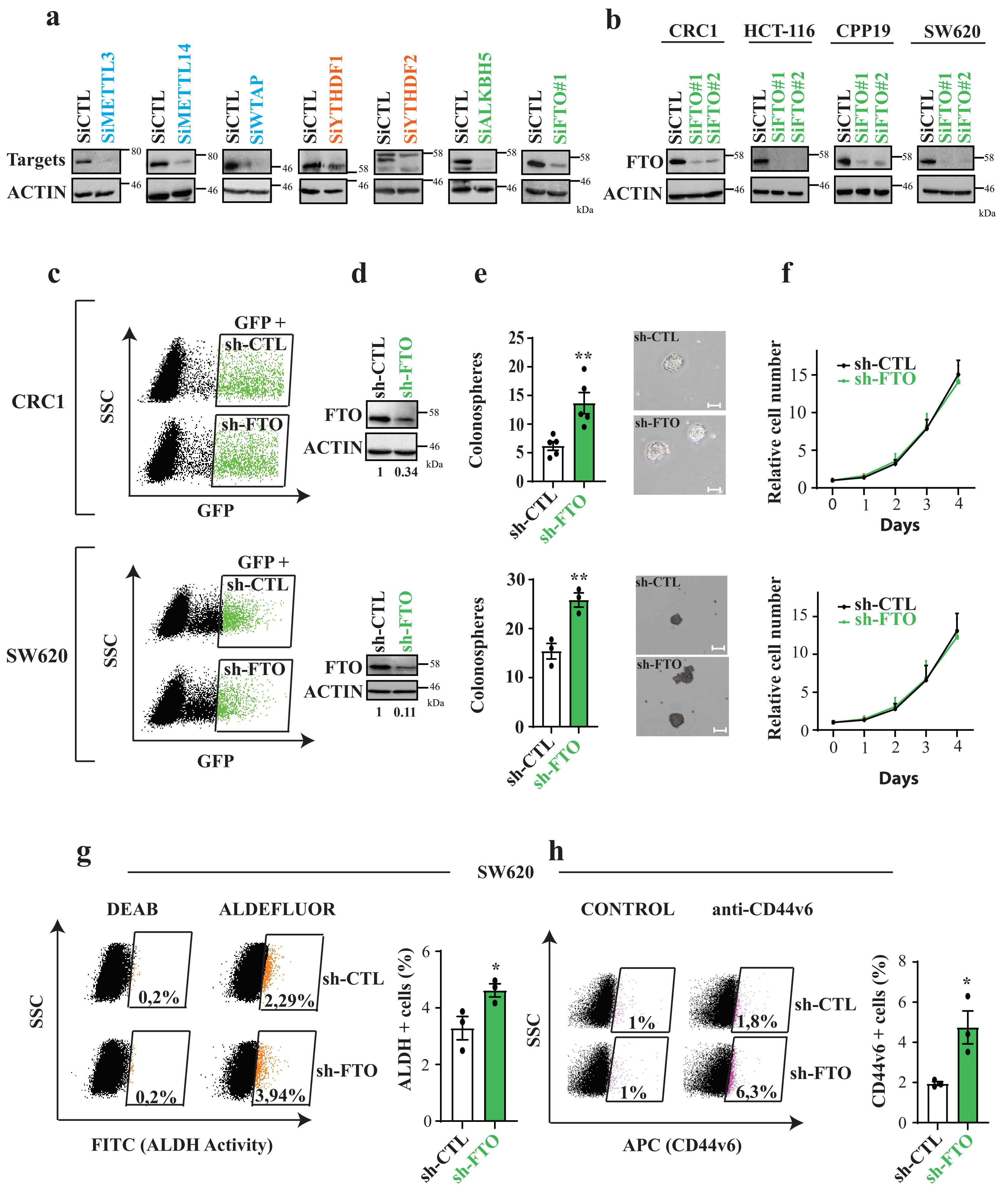

a
SiCTL
SiMETTL3
SiMETTL14
SiWTAP
SiYTHDF1
SiYTHDF2
SiALKBH5
SiFTO#1
Targets
ACTIN
kDa
b
CRC1
HCT-116
CPP19
SW620
SiFTO#2
FTO
c
CRC1
SW620
GFP +
sh-CTL
sh-FTO
SSC
GFP
d
1 0.34
1 0.11
e
Colonospheres
f
Relative cell number
Days
g
h
DEAB
ALDEFLUOR
0,2%
2,29%
3,94%
FITC (ALDH Activity)
ALDH + cells (%)
CONTROL
anti-CD44v6
1%
1,8%
6,3%
APC (CD44v6)
CD44v6 + cells (%)

Figure S1. (a-b) Depletion of m^{6}A actors by siRNA. (a) Western blot analysis was performed to check the depletion of the m6A -related proteins. Representative of three independant experiments. **(b) Control of FTO depletion in 4 cell lines.** Pictures are representative of three experiments. **(c - d)** Establishment of stable cell lines. (c) Efficiently infected cells by sh-RNA retrovirus were sorted by FACS on the basis of GFP expression (CRC1 top, SW620 bottom). **(d)** Efficient FTO depletion was evaluated by immunoblot. **(e) Stable FTO silencing increases sphere forming potential of CRC cell lines.** Colonosphere number after FTO silencing in CRC1 (top) and SW620 (bottom) cell lines. Results represent mean +/- S.E.M of five experiments for CRC1 and three experiments for SW620. ** p-value < 0.01, Two-sided Unpaired T-test. **(f) FTO silencing does not affect cell proliferation.** Cell proliferation was measured every day for 4 days using sulforhodamine assay (CRC1 top, SW620 bottom). **(g–h) Stable FTO silencing increases CSC markers in SW620 cell line.** Number of ALDH (left) and CD44v6 (right) positive cells were assessed by Flow cytometry. Results are mean +/- S.E.M of three experiments. * p-value < 0.05, Two-sided Unpaired T-test.

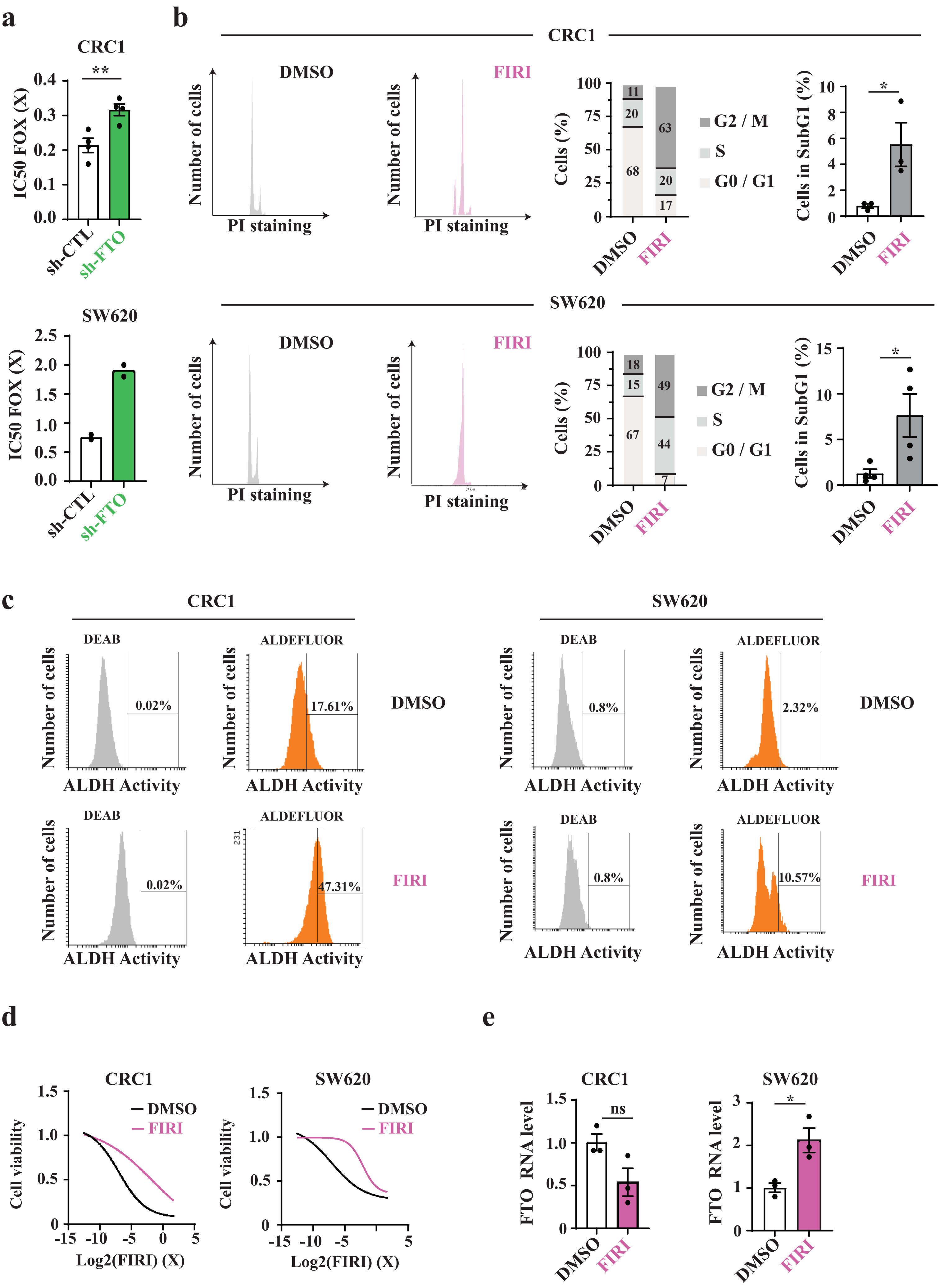
a
CRC1
IC50 FOX (X)
**
sh-CTL
sh-FTO
SW620
b
CRC1
DMSO
FIRI
Number of cells
PI staining
Cells (%)
11
20
68
63
20
17
G2 / M
S
G0 / G1
Cells in SubG1 (%)
*
SW620
18
15
67
49
44
7
c
CRC1
SW620
DEAB
ALDEFLUOR
Number of cells
ALDH Activity
0.02%
17.61%
47.31%
0.8%
2.32%
10.57%
DMSO
FIRI
d
CRC1
SW620
Cell viability
Log2(FIRI) (X)
DMSO
FIRI
e
FTO RNA level
ns
*

Figure S2. (a) FTO silencing increases resistance to FOX. Cell viability was measured upon exposure of cells to increasing dose of FOX (1 X = 50 µM 5-FU and 1 µM of oxaliplatin) in CRC1 (top) and SW620 (bottom). Results represent mean +/- S.E.M of four experiments (CRC1) or two experiments representative of three (SW620). ** p-value < 0.01, Two-sided Unpaired T-test. **(b) FIRI treatment blocks cell cycle in G2/M.** Cell cycle distribution was analyzed after FIRI pre-treatment at 0.2 X for 72 h in CRC1 (top) and SW620 (bottom). Cell events occurring before G0/G1 peak were considered as subG1. Bar plots represent mean +/- S.E.M of three (CRC1) and four experiments (SW620). *p-value < 0.05, Two-sided unpaired T-test. Gating strategy is illustrated in Figure S8f and S8g **(c) FIRI treatment enriches cell population in ALDH positive cells.** Number of ALDH positive cells after 72 h of 0.2 X FIRI treatment in CRC1 (left) and SW620 (right). Representative of three biological replicates. Gating strategy is shown in Figure S8h and S8i **(d) FIRI treatment selects chemoresistant cells.** Measurement of FIRI toxicity on CRC1 (left) and SW620 (right) pre-treated with 0.2 X of FIRI for 72 h. Cell viability was measured upon exposure of cells to increasing dose of FIRI as in 2a after a first round of 0.2X FIRI pre-treatment for 72 h. Results show one experiment representative of three. **(e) FIRI treatment impact FTO transcripts level in a cell type dependent manner.** Transcript level of FTO was analyzed by RT-qPCR after 72 h of FIRI treatment at 0.2X in CRC1 (left) and SW620 (right). Bar plot represent mean +/- S.E.M of three biological replicates. ns = non significant, *p-value < 0.05, Two-sided unpaired T-test.

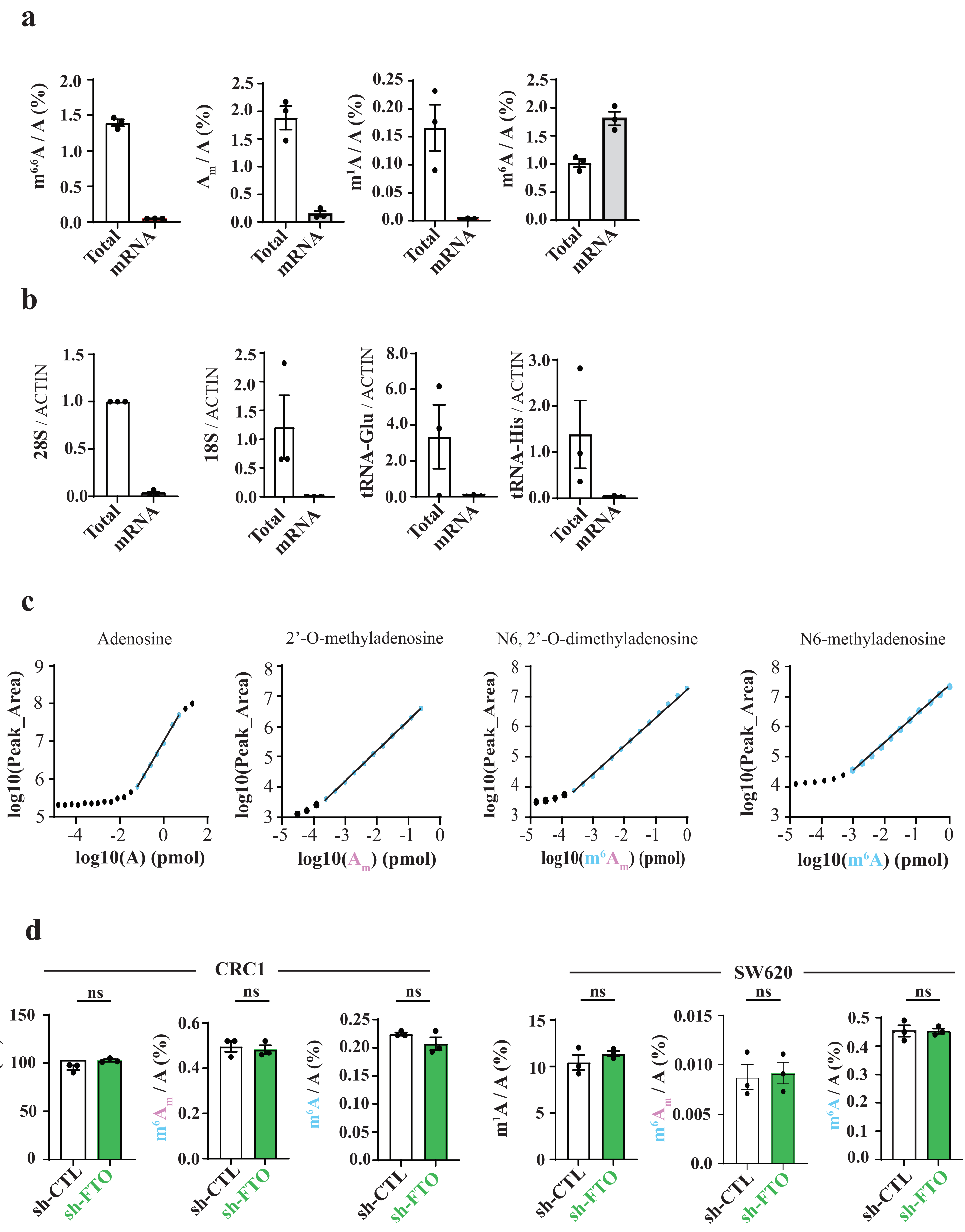
a
m6,6A / A (%)
Am / A (%)
m1A / A (%)
m6A / A (%)
Total
mRNA
b
28S / ACTIN
18S / ACTIN
tRNA-Glu / ACTIN
tRNA-His / ACTIN
Total
mRNA
c
Adenosine
2'-O-methyladenosine
N6, 2'-O-dimethyladenosine
N6-methyladenosine
log10(Peak_Area)
log10(A) (pmol)
log10(Am) (pmol)
log10(m6Am) (pmol)
log10(m6A) (pmol)
d
CRC1
SW620
ns
m1A / A (%)
m6Am / A (%)
m6A / A (%)
sh-CTL
sh-FTO

Figure S3. (a) tRNA- and rRNA-enriched modifications sharply decrease upon mRNA purification. Highly prevalent modifications in tRNA (m^1A) and rRNA ($m^{6,6}A$; A_m) or mRNA (m^6A) were quantified by mass-spectrometry from total RNA and polyA+ mRNA extracted from SW620 cell line to control for mRNA enrichment. Bar plots represent mean +/- S.E.M of three biological replicates. **(b) rRNA and tRNA level collapse after mRNA purification**. 28 S and 18 S rRNA, tRNA-Glu and tRNA-His levels were quantified by RT-PCR from "Total" RNA and polyA + "mRNA" extracted from SW620 cell line. Bar shows mean +/- S.E.M of 3 biological replicates. **(c) Quantifications of mRNA modifications by LC-MS/MS.** Standard curve of nucleosides standard A, m^6A_m, Am and m^6A. **(d) FTO silencing does not affect small RNA modifications.** Small RNA were purified from sh-FTO and sh-CTL cell line (CRC1 (left) and SW620 (right)) and RNA modifications were quantified by LC-MS/MS. Bar plots represent mean +/- S.E.M of three independent experiments. ns = not significant. Two sided Unpaired T-test.

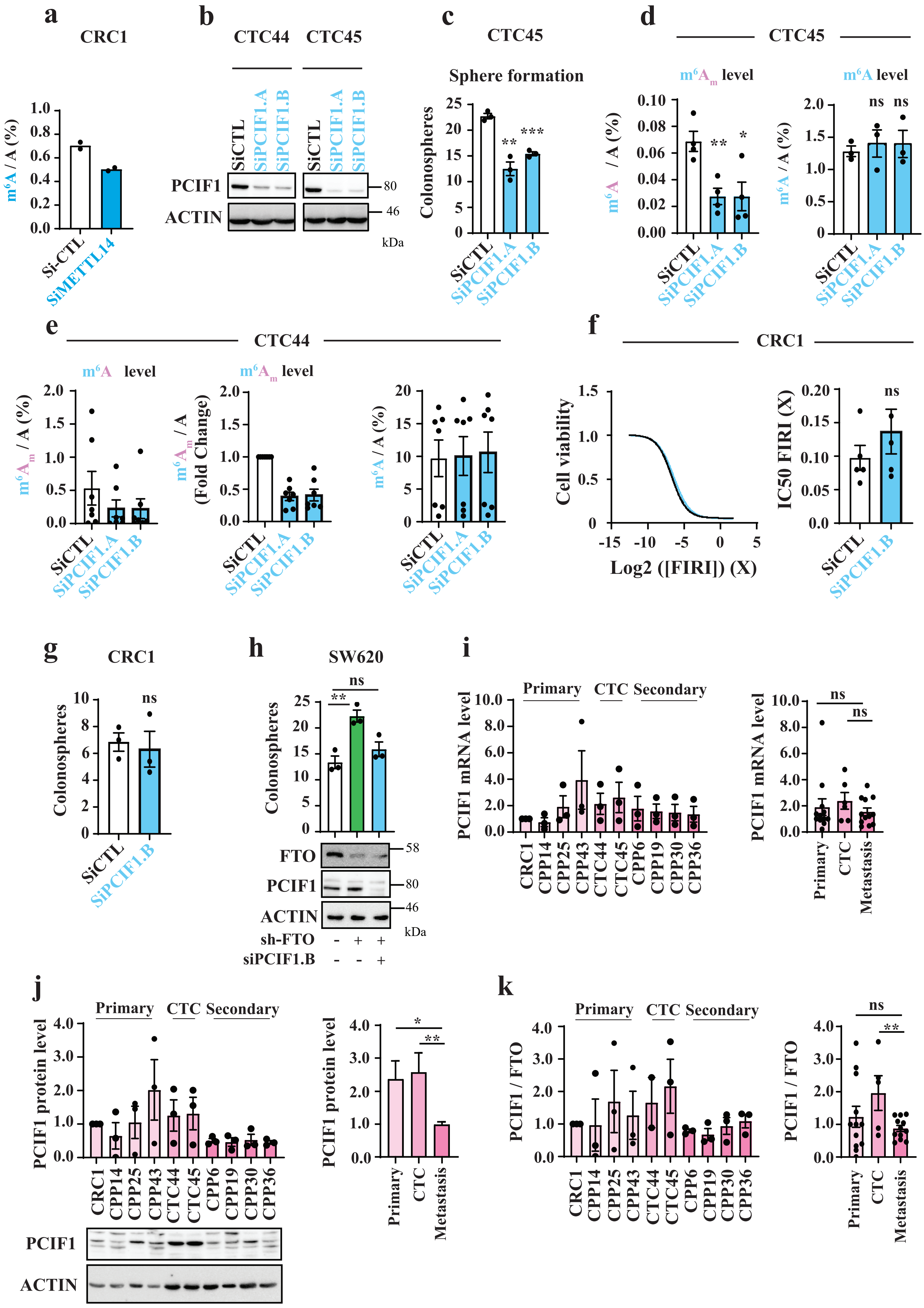

a
CRC1
b
CTC44
CTC45
c
CTC45
Sphere formation
d
CTC45
e
CTC44
f
CRC1
g
CRC1
h
SW620
i
j
k

Figure S4. (a) METTL14 knockdown significantly decrease m6A level. m6A level of polyA + mRNA was analyzed by mass-spectrometry in CRC1 cell line. Result represents mean +/- S.E.M of two experiments. *p-value < 0.05. Two sided Unpaired T-test **(b) Control of PCIF1 depletion.** PCIF1 expression was evaluated by immunoblot upon siRNA treatment in CTCs cell lines (CTC44 (left), CTC45 (right)). **(c) PCIF1 knockdown decreases sphere formation.** Sphere formation assay was performed after siRNA treatment targeting PCIF1 in CTC45 cell line. Bar plot represent mean +/- S.E.M of at least 3 biological replicates. *** p-value < 0.001, ** p-value < 0.01. Two sided unpaired T-test **(d) PCIF1 silencing decreases m6Am level in mRNA.** Quantification of polyA+ mRNA modification by mass-spectrometry upon silencing of PCIF1 in CTC45 cell line. Bar plot represents mean +/- S.E.M of at least three biological replicates. **p-value < 0.01, * p-value < 0.05, ns = not significant, Two-sided Unpaired T-test. **(e) PCIF1 knockdown decreases m6Am level in mRNA.** Quantification of polyA+ mRNA modification by mass-spectrometry upon silencing of PCIF1 in CTC44 cell line. Bar plot represents mean +/- S.E.M of seven biological replicates. **p-value < 0.01, * p-value < 0.05 Two-sided Unpaired T-test. **(f) PCIF1 knockdown does not affect resistance to FIRI treatment in CRC1.** Chemosensitivy to FIRI was assessed by treating siRNA treated CRC1 cell line with decreasing dose of drugs. IC50 was determined graphically. Bar plot represents mean +/- S.E.M of four biological replicates. ns = not significant, two-sided Unpaired T-test. **(g) PCIF1 knockdown does not affect sphere-forming ability in CRC1.** Sphere formation assay was performed after siRNA treatment targeting PCIF1 in CRC1 cell line. Bar plot represent mean +/- S.E.M of 3 biological replicates. ns = not significant, two-sided Unpaired T-test. **(h) PCIF1 knockdown rescues basal sphere formation in sh-FTO cell line.** Sphere formation was performed upon depletion of PCIF1 by siRNA treatment of both sh-CTL and sh-FTO SW620 cell lines. Bar plot represent mean +/- S.E.M of 3 biological replicates. Proteins depletion were assessed by western blot. **p-value < 0.01, ns = not significant. One way Anova followed by multiple comparisons to sh-CTL. **(i) PCIF1 mRNA level does not change along with tumor progression.** PCIF1 mRNA level were analyzed by RT-PCR in various colon cancer cell lines that derived from primary tumor, liquid biopsies (CTC) and secondary tumor. Bar plot represent mean +/- S.E.M of PCIF1 transcript level per cell line or per group. ns = not significant, One way-Anova. **(j) PCIF1 protein level is decreased in metastatic cell line.** PCIF1 protein was quantified in patient derived cell line (same as in S5a) by western blot. Bar plot represents mean +/- S.E.M per cell line or per group. **p-value < 0.01, ns = not significant. One-way Anova followed by multiple comparisons. **(k) The ratio PCIF1 / FTO is only increased in CTCs.** Quantification of the ratio of PCIF1 protein level and FTO protein level by western blot. Bar plot represents mean +/- S.E.M of three biological replicates.

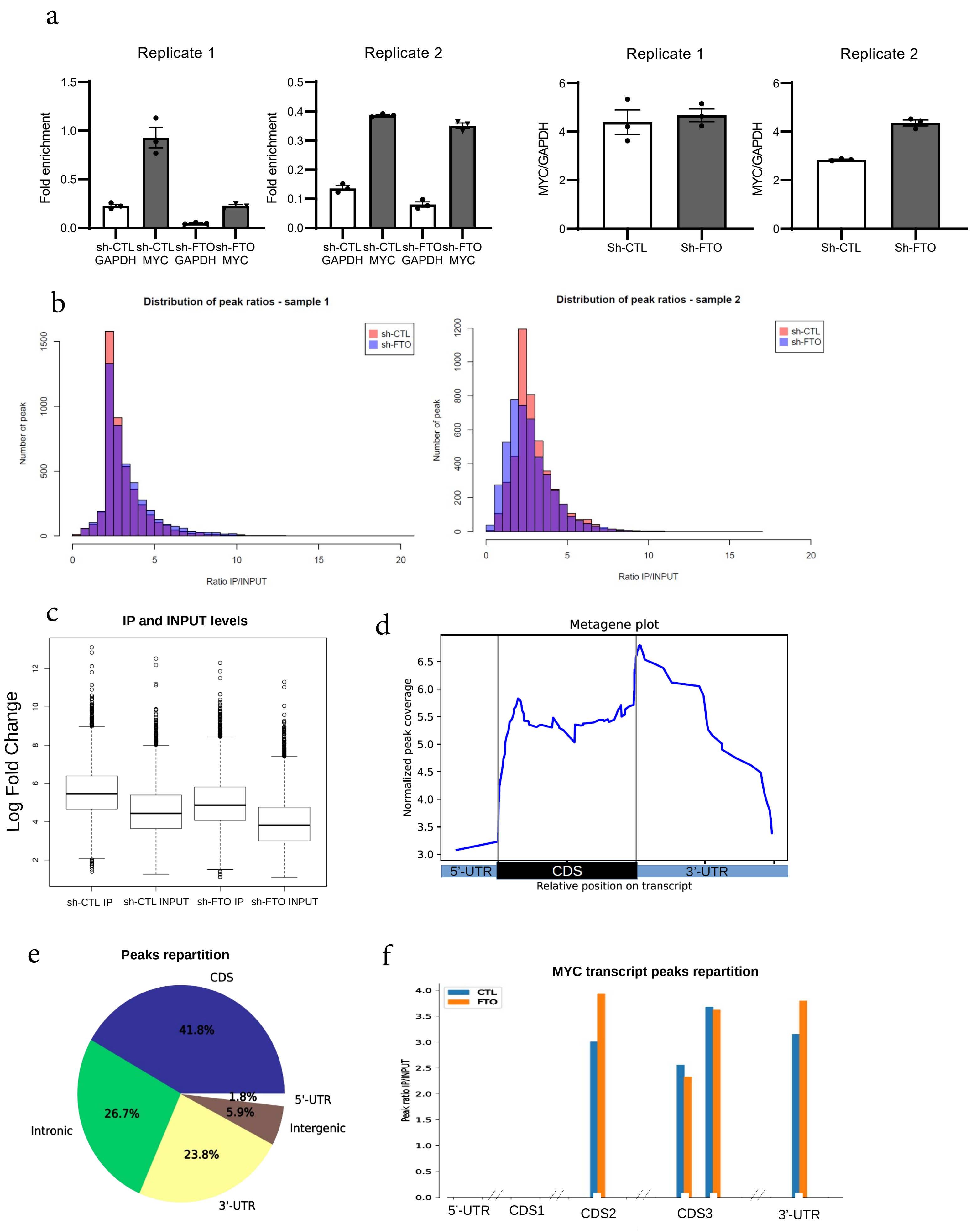
a
Replicate 1
Replicate 2
Fold enrichment
sh-CTL GAPDH
sh-CTL MYC
sh-FTO GAPDH
sh-FTO MYC
MYC/GAPDH
Sh-CTL
Sh-FTO
b
Distribution of peak ratios - sample 1
Distribution of peak ratios - sample 2
Number of peak
Ratio IP/INPUT
sh-CTL
sh-FTO
c
IP and INPUT levels
Log Fold Change
sh-CTL IP
sh-CTL INPUT
sh-FTO IP
sh-FTO INPUT
d
Metagene plot
Normalized peak coverage
5'-UTR
CDS
3'-UTR
Relative position on transcript
e
Peaks repartition
CDS
41.8%
1.8%
5'-UTR
5.9%
Intergenic
26.7%
Intronic
23.8%
3'-UTR
f
MYC transcript peaks repartition
CTL
FTO
Peak ratio IP/INPUT
5'-UTR
CDS1
CDS2
CDS3
3'-UTR

Figure S5. (a) Quality control of MeRIP-seq procedure by RT-qPCR. In the left panel, m6A immunoprecipitation (IP) is normalized by the input. In the right panel, m6A IP at MYC transcript is normalized by the input and represented relative to GAPDH. We have used MYC and GAPDH transcripts as positive and negative controls respectively. Bar plots represent mean +/- S.E.M of three technical replicates. N=2 biological replicates. **(b) Distribution of peak ratio IP/INPUT for replicate 1 and replicate 2 sh-CTL and sh-FTO.** Specific parts corresponding to detected peak are colored in red for sh-CTL and in blue for sh-FTO; common parts are colored in violet (red +blue). **(c) Boxplot of IP and INPUT levels for both replicates of sh-CTL and sh-FTO.** Each box represents the dispersion of log fold change level. The boundary of the box closest to zero indicates the 25th percentile, a black line within the box marks the median and the boundary of the box farthest from zero indicates the 75th percentile. Whiskers above and below the box indicate the 10th and 90th percentiles. IP samples are enriched in comparison to INPUT samples.IP samples are enriched in comparison to INPUT samples. N=2. **(d)** Metagene plot illustrating m6A peak repartition for all identified genes within coding regions (CDS), 5'-UTR and 3'-UTR (e) Percentage of m6A peak repartition for all identified genes, with CDS, 5'-UTR, 3'-UTR, intronic and intergenic parts. (f) m6A peak repartition for MYC gene for sh-CTL and sh-FTO along the transcript, comprising 5'-UTR, 3 CDS and 3'-UTR. N=2.

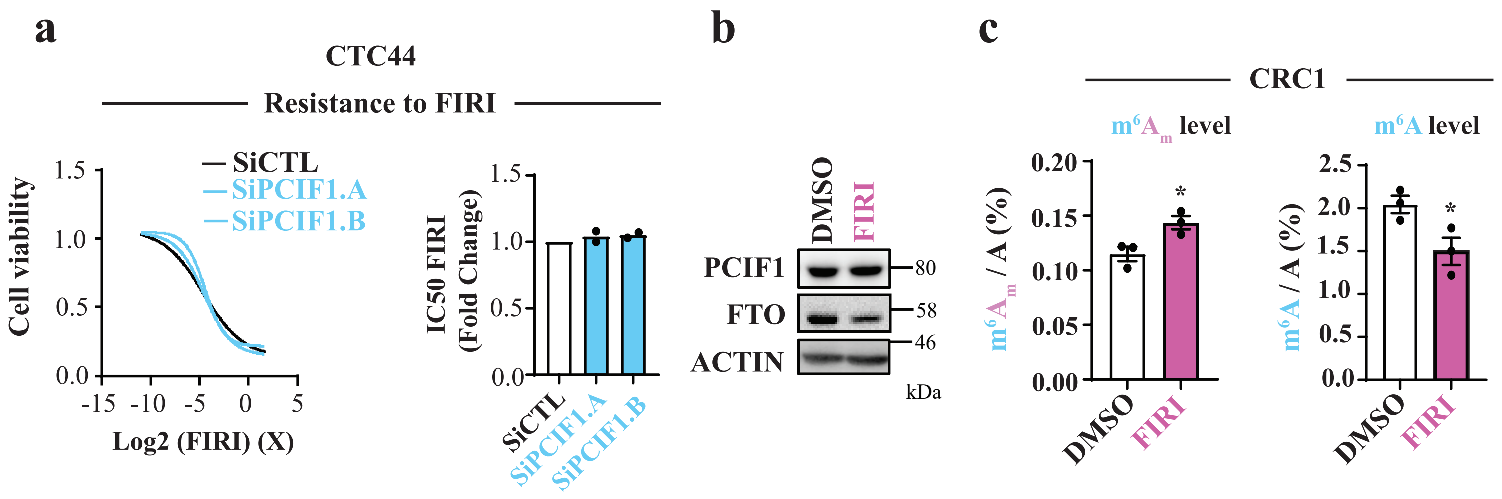

Figure S6. (a) PCIF1 silencing does not impact chemoresistance in vitro. Chemosensitivy to FIRI was assessed by treating siRNA treated CTC44 cell line with decreasing dose of drugs. IC50 was determined graphically. Bar plot represents mean of two biological replicates. **(b) PCIF1 level is unchanged upon FIRI treatment.** Analysis of FTO and PCIF1 level by western blot after cell treatment by FIRI 0.2 X for 72 h in CRC1 cell line. Picture of FTO is the same as in Figure 2d and picture of PCIF1 is representative of three biological replicates. **(c) FIRI treatment increases m6Am level.** m6Am / A and m6A / A level were assessed by LC-MS/MS on polyA + mRNA from CRC1 cells either treated with 0.2X of FIRI for 72 h or treated with DMSO. Bar plots represent mean +/- S.E.M of three biological replicates. *p-value < 0.05, ns = not significant, Two-sided Unpaired T-test.

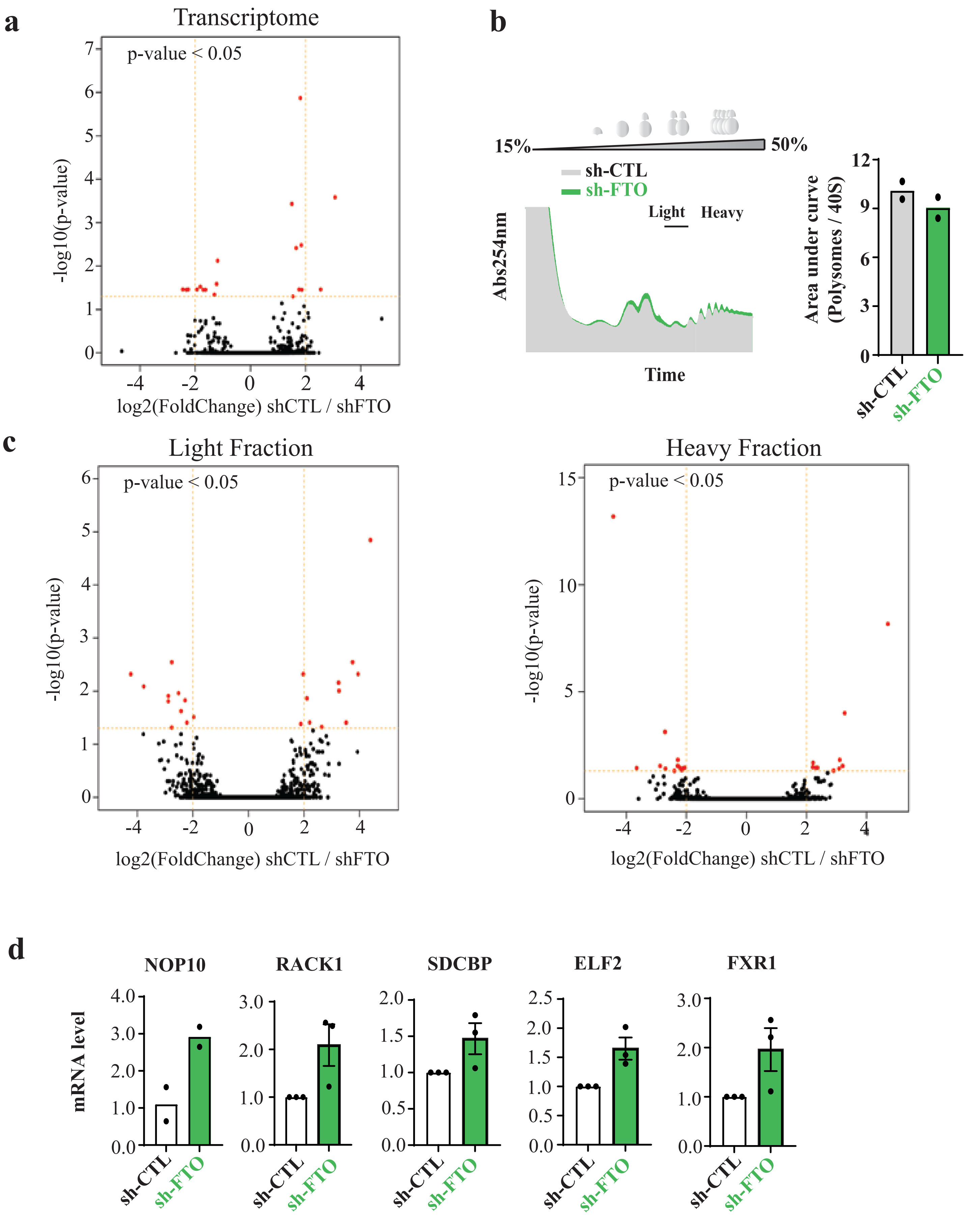
a
Transcriptome
p-value < 0.05
-log10(p-value)
log2(FoldChange) shCTL / shFTO
b
15%
50%
sh-CTL
sh-FTO
Light
Heavy
Abs254nm
Time
Area under curve
(Polysomes / 40S)
sh-CTL
sh-FTO
c
Light Fraction
p-value < 0.05
-log10(p-value)
log2(FoldChange) shCTL / shFTO
Heavy Fraction
p-value < 0.05
-log10(p-value)
log2(FoldChange) shCTL / shFTO
d
NOP10
RACK1
SDCBP
ELF2
FXR1
mRNA level
sh-CTL
sh-FTO

Figure S7. FTO silencing does not trigger significant change of gene expression level. (a) FTO silencing does not affect global transcript level. MA plot analyses of transcripts changes. Red dot represent transcripts with an adjusted p-value < 0.05. Pie Chart shows the percentage of transcripts with fold change > 2 or < 0.5; CRC1 line n = 3 individual experiments. **(b) FTO does not affect global translation activity. Polysome profile of sh-CTL and sh-FTO CRC1 cell lines.** Light fraction (1 to 3 ribosomes/mRNA) and heavy fractions are indicated. Bar plot represents polysome level quantification of three experiments. N=2 biological replicates. **(c) FTO silencing triggers very mild change of translation of specific mRNA.** MA plot analyses of transcripts changes in Light and Heavy fractions. Same representation as in (a); CRC1 line, n = 3. **(d) Validation of differentially expressed gene in translatome data.** RT-PCR were performed on heavy fraction. Results show mean ± S.E.M of three biological replicates or two representative of three (NOP10). For each gene, cycle threshold (ct) was normalized to ct of ACTIN. Dct were further normalized to dct of sh-CTL condition. *p-value < 0.05, Two sided Unpaired T-test.

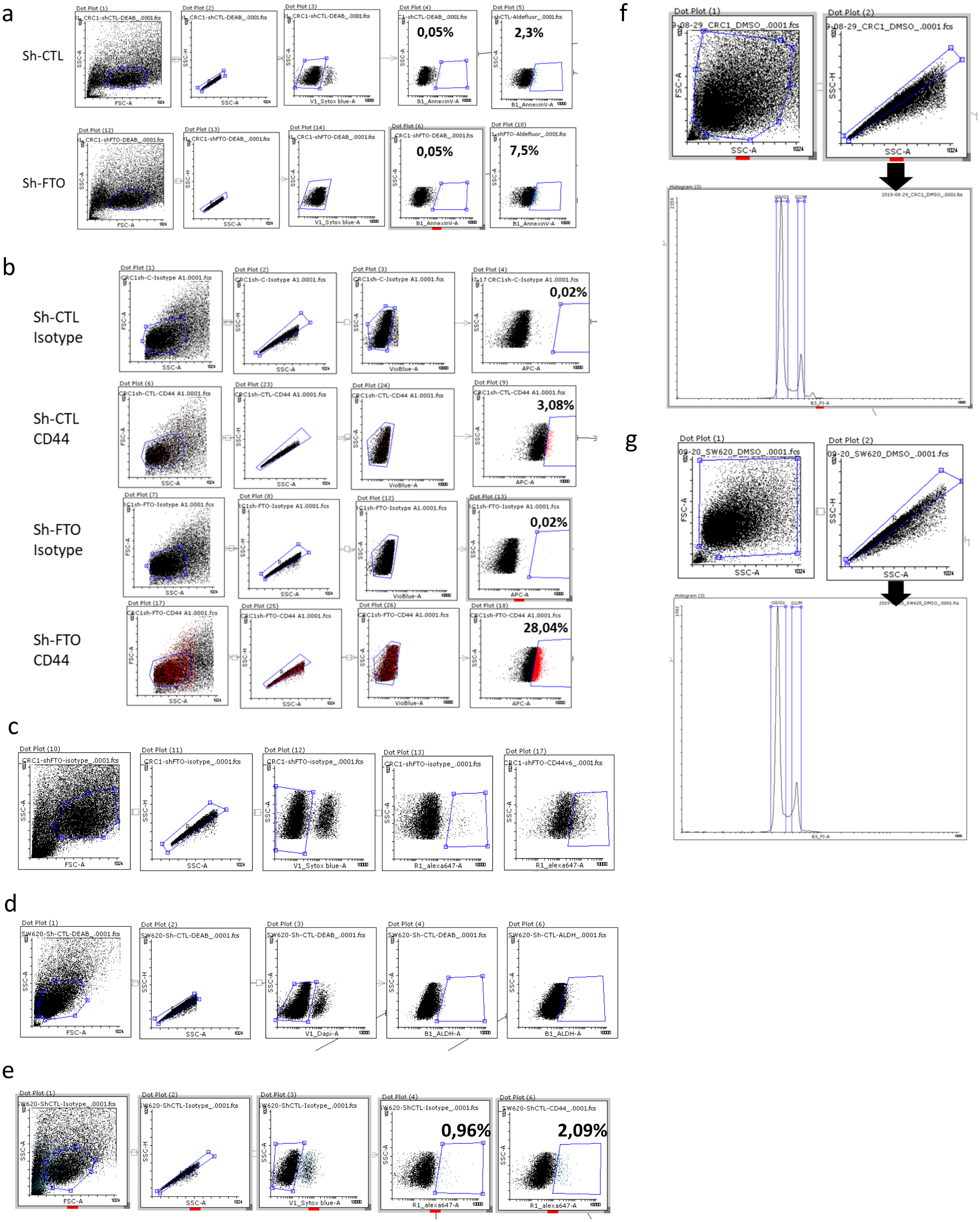
a
Sh-CTL
Sh-FTO
0,05%
2,3%
0,05%
7,5%
b
Sh-CTL Isotype
Sh-CTL CD44
Sh-FTO Isotype
Sh-FTO CD44
0,02%
3,08%
0,02%
28,04%
c
d
e
0,96%
2,09%
f
g
Dot Plot (1)
Dot Plot (2)
FSC-A
SSC-A
SSC-H
G0/G1
G2/M

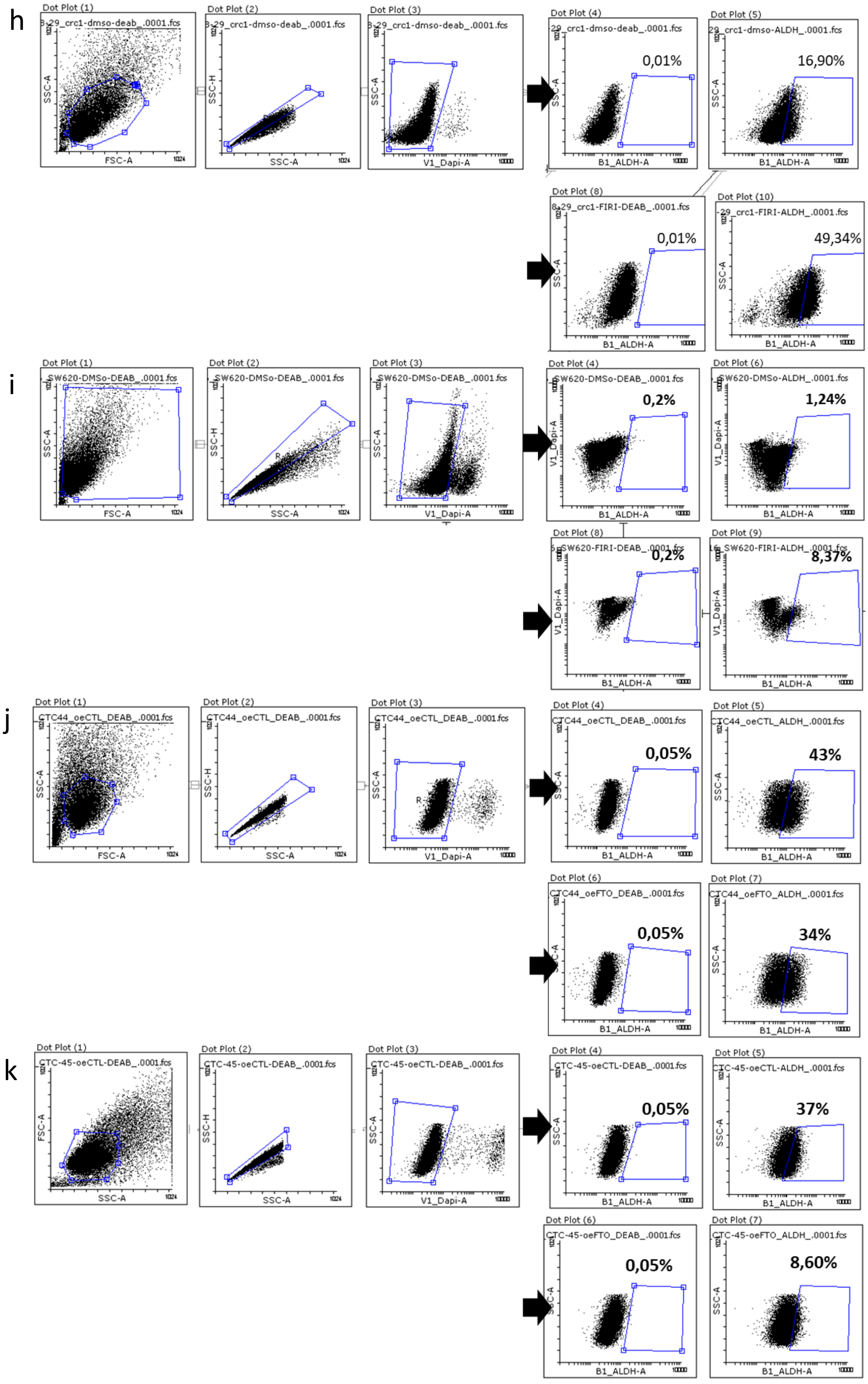

Figure S8. Gating strategies. (a) ALDH staining of CRC1 sh-CTL and shFTO ALDH cells (Figure 1e). **(b)** CD44 staining of CRC1 sh-CTL vs shFTO cells (Figure 1e). **(c)** CD44v6 staining of CRC1 sh-CTL vs shFTO cells (Figure 1e). **(d)** ALDH staining of SW620 (Figure S1g). **(e)** CD44 staining of SW620 sh-CTL vs shFTO cells (Figure S1h). **(f)** PI staining of CRC1 (Figure S2b). **(g)** PI staining of SW620 (Figure S2c). **(h)** ALDH staining of CRC1 DMSO vs CRC1 FIRI (Figure S2c). **(i)** ALDH staining of SW620 DMSO vs SW620 FIRI (Figure S2c). **(j)** ALDH staining of CTC44 oe-CTL (Figure 3e). **(k)** ALDH staining of CTC45 oe-CTL (Figure 3e).

Table S1: list of primers

Primers	Sequence 5' – 3 '
FTO-Forward	ACTTGGCTCCCTTATCTGACC
FTO-Reverse	TGTGCAGTGTGAGAAAGGCTT
ACTIN-Forward	AGCACGGCATCGTCACCAACT
ACTIN-Reverse	TGGCTGGGGTGTTGAAGGTCT
NOP10-Forward	AAACGCTTCAAGGTGCTCAT
NOP10-Reverse	ACCATGATTGCCTCACACAA
RACK1-Forward	TGAGTGTGGCCTTCTCCTCT
RACK1-Reverse	GCTTGCAGTTAGCCAGGTTC
SDCBP-Forward	TTCTGCTCCTATCCCTCACG
SDCBP-Reverse	CCAGTTACAGGAGCCACCAT
ELF2-Forward	AGGGGAATTCTTGCAAAGGT
ELF2-Reverse	ACACCCTTCTCTGCTCTGGA
PIAS1-Forward	CCTGGGTTTGTCCTGTCTGT
PIAS1-Reverse	AGCTCAAGCATCCATCGACT
28S-Forward	CGACGTTGCTTTTTGATCCT
28S-Reverse	GCAACGACAAGCCATCAGTA
18S-Forward	TGTGCCGCTAGAGGTGAAATT
18S-Reverse	TGGCAAATGCTTTCGCTTT
tRNAGlu-Forward	CCTGGTGGTCTAGTGGCTAGGA
tRNAGlu-Reverse	TCCCTGACCGGGAATCGAA
tRNAHis-Forward	GCC GTGATCGTATAGTGGT
tRNAHis-Reverse	TGACTCGGATTCGAACCGA
GAPDH-Forward	TGGTATCGTGGAAGGACTCA
GAPDH-Reverse	CCAGTAGAGGCAGGGATGAT
MYC-Forward (m6A peak in 3'UTR)	CATCAGCACAACTACGCAGC
MYC- Reverse (m6A peak in 3'UTR)	GCTGGTGCATTTTCGGTTGT

Table S2: list of antibodies

Antibodies	Manufacturers	References	Dilution
Anti-METTL3	Abnova	H00056339-B01P	1 : 500
Anti-METTL14	Sigma	HPA038002	1 : 1,000
Anti-WTAP	Santa-Cruz	Sc-374280	1 : 300
Anti-YTHDF1	Abcam	ab99080	1 : 500
Anti-YTHDF2	ProteinTech	24744-1-AP	1 : 500
Anti-ALKBH5	Sigma	HPA007196	1 : 1,000
Anti-FTO	Phosphosolution	597-FTO	1 : 1,000
Anti-FTO	AbCam	EPR6895	1: 1, 000
Anti-PCIF1	ProteinTech	16082-1-AP	1 : 1,000
Anti-ACTIN	Sigma	A5441	1 : 20,000
Anti-CD44	BD biosciences	59942	1 : 500
Anti-CD44v6	Myltenyi Biotec	130-111-238	1 : 300
anti-IgG2a	BD biosciences	130-091-836	1 : 500
REA-S control Isotype	Myltenyi Biotec	130-104-614	1 : 300
Anti-mouse IgG	Santa-Cruz	Sc-516102	1 : 10,000
Anti-Rabbit IgG	Cell Signaling	7074S	1 : 1,000

Tables S3: list of si-RNAs

Target	si-RNA sequence 5' – 3 '
si-METTL3	CUGCAAGUAUGUUCACUAUGA
si-METTL14	AAGGAUGAGUUAAUAGCUAAA
si-WTAP	AAGCUUUGGAGGGCAAGUACA
si-YTHDF1	CCGCGUCUAGUUGUUC AUGAA
si-YTHDF2	CAGGCUGGAGAAUAACGACAA
si-ALKBH5	ACAAGUACUUCUUCGGCGATT
si-FTO#1	AAAUAGCCGCUGCUUGUGAGA
si-FTO#2	ACGAAUUGCCCGAACAUUA

Table S4: significantly deregulated genes in transcriptome (sh-CTL vs sh-FTO)

ensembl_transcript_id	ensembl_gene_id	hgnc_symbol	baseMean	log2FoldChange	padj
ENST00000379284.1	ENSG00000145990	GFOD1	78,77927178	-2,45	3,45E-02
ENST00000502871.5	ENSG00000138756	BMP2K	68,55905687	-2,32	3,50E-02
ENST00000560346.5	ENSG00000137843	PAK6	70,25170115	-2,25	3,45E-02
ENST00000613981.3	ENSG00000276016	ABR	345,208247	-1,93	3,45E-02
ENST00000553804.5	ENSG00000135424	ITGA7	55,07126719	-1,82	2,98E-02
ENST00000469698.5	ENSG00000141698	NT5C3B	79,73094196	-1,71	3,50E-02
ENST00000539396.5	ENSG00000090372	STRN4	53,85657803	-1,63	3,50E-02
ENST00000423729.2	ENSG00000185818	NAT8L	363,546431	-1,30	4,55E-02
ENST00000415298.5	ENSG00000152056	AP1S3	173,4381645	-1,23	2,59E-02
ENST00000296978.3	ENSG00000164484	TMEM200A	308,8592893	-1,19	7,52E-03
ENST00000268349.7	ENSG00000140718	FTO	273,6964792	1,14	0,06
ENST00000358075.10	ENSG00000102158	MAGT1	948,4366512	1,50	3,71E-04
ENST00000540441.6	ENSG00000166783	MARF1	77,00415504	1,54	5,00E-02
ENST00000453408.7	ENSG00000141503	MINK1	106,3068925	1,66	3,82E-03
ENST00000635816.1	ENSG00000107779	BMPR1A	236,814228	1,76	3,45E-02
ENST00000369409.9	ENSG00000092621	PHGDH	346,5642954	1,81	1,351E-06
ENST00000508376.6	ENSG00000134982	APC	316,6998041	1,85	3,28E-03
ENST00000551775.5	ENSG00000196531	NACA	35,05782626	1,86	3,50E-02
ENST00000645393.1	ENSG00000197467	COL13A1	27,78249527	2,55	3,45E-02
ENST00000620953.2	ENSG00000274081	PUF60	141,1437039	3,06	2,60E-04

Table S5: significantly deregulated genes in light polysome (sh-CTL vs sh-FTO)

ensembl_transcript_id_gene	ensembl_gene_id	hgnc_symbol	BaseMean	log2FoldChange	padj
ENST00000473095,1	ENSG00000106153	CHCHD2	259,73024	-4,23	0,00476437
ENST00000573001,5	ENSG00000006327	TNFRSF12A	265,6137962	-3,77	0,0081455
ENST00000511295,1	ENSG00000164109	MAD2L1	115,7058686	-2,88	0,01551306
ENST00000396296,7	ENSG00000136758	YME1L1	84,88862546	-2,88	0,01228001
ENST00000540297,6	ENSG00000166598	HSP90B1	109,8066838	-2,76	0,04842021
ENST00000546921,1	ENSG00000170421	KRT8	58,98741964	-2,76	0,0028436
ENST00000426663,1	ENSG00000117475	BLZF1	64,45441125	-2,52	0,01087912
ENST00000578758,5	ENSG00000108654	DDX5	180,4850595	-2,42	0,02370278
ENST00000440865,2	ENSG00000153187	HNRNPU	260,0176582	-2,28	0,01486871
ENST00000531140,1	ENSG00000109929	SC5D	96,40538527	-2,22	0,03923588
ENST00000583036,5	ENSG00000265681	RPL17	1353,617173	-1,97	0,03056144
ENST00000472729,1	ENSG00000173163	COMMD1	147,8102686	1,89	0,04127679
ENST00000395577,2	ENSG00000100526	CDKN3	615,3449577	1,98	0,00476437
ENST00000442660,5	ENSG00000159147	DONSON	132,045702	2,10	0,01363747
ENST00000614778,4	ENSG00000249915	PDCD6	536,122248	2,20	0,03899492
ENST00000361166,8	ENSG00000186575	NF2	151,0018483	2,64	0,04710098
ENST00000426073,6	ENSG00000102606	ARHGEF7	243,2549723	3,24	0,00688657
ENST00000515539,5	ENSG00000145781	COMMD10	71,24704077	3,26	0,00983477
ENST00000483423,5	ENSG00000223766	PRR3	35,70491387	3,52	0,03923588
ENST00000487302,3	ENSG00000233564	PRR3	35,70491387	3,52	0,03923588
ENST00000529725,1	ENSG00000213445	SIPA1	35,922234	3,75	0,0028436
ENST00000582770,6	ENSG00000105655	ISYNA1	117,7625859	3,95	0,00476142
ENST00000604351,5	ENSG00000196422	PPP1R26	142,5128676	4,39	1,42E-05

Table S6: significantly deregulated genes in heavy polysome (sh-CTL vs sh-FTO)

ensembl_transcript_id	ensembl_gene_id	hgnc_symbol	baseMean	log2FoldChange	padj
ENST00000557912,1	ENSG00000182117	NOP10	426,0909514	-4,43	6,56E-14
ENST00000507000,5	ENSG00000204628	RACK1	169,8339352	-3,66	0,036224085
ENST00000468861,5	ENSG00000114416	FXR1	334,0809707	-2,88	0,028827874
ENST00000522610,5	ENSG00000070501	POLB	219,1002589	-2,71	0,000743499
ENST00000424270,6	ENSG00000137575	SDCBP	255,1401181	-2,70	0,03835289
ENST00000577650,5	ENSG00000141526	SLC16A3	280,8281468	-2,40	0,049456175
ENST00000315741,5	ENSG00000122406	RPL5	1106,973304	-2,29	0,028827874
ENST00000373632,8	ENSG00000118705	RPN2	57,33589018	-2,29	0,015093056
ENST00000544233,5	ENSG00000176871	WSB2	66,42820584	-2,28	0,028827874
ENST00000438908,5	ENSG00000230230	TRIM26	163,5899189	-2,16	0,045213002
ENST00000578804,5	ENSG00000108654	DDX5	135,056588	-2,10	0,036224085
ENST00000319980,10	ENSG00000032742	IFT88	57,39826553	-2,05	0,035151201
ENST00000593027,6	ENSG00000105677	TMEM147	231,1540574	2,21	0,033499831
ENST00000472481,5	ENSG00000142541	RPL13A	3916,00591	2,22	0,020845154
ENST00000545237,1	ENSG00000033800	PIAS1	48,03035127	2,31	0,035151201
ENST00000442386,5	ENSG00000143727	ACP1	974,4346148	2,37	0,035151201
ENST00000568146,1	ENSG00000140939	NOL3	119,4543559	2,90	0,048460803
ENST00000394235,6	ENSG00000109381	ELF2	31,63957441	3,08	0,03835289
ENST00000518015,5	ENSG00000132912	DCTN4	24,28381223	3,11	0,015093056
ENST00000491937,6	ENSG00000171863	RPS7	1403,902944	3,20	0,028827874
ENST00000527361,5	ENSG00000121753	ADGRB2	68,93376841	4,71	6,80E-09

Table S7: HOMER results: the three most significant motif detected by Homer on the sequences of MERIP peaks. The first motif contains the DRACH motif for m6A sites.

Rk	Motif	P-val	Info. Content	Nb targets	% Targets	% Background	Motif Logo
1	RWGGACT	1e-78	1.828	2045	59.46%	43.47%	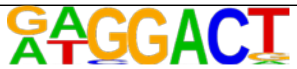
2	GTTCTTC	1e-58	1.572	2191	63.71%	49.97%	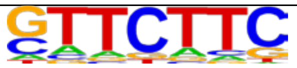
3	CATCGTC	1e-48	1.743	1215	35.33%	24.08%	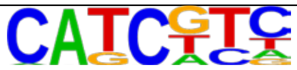

Table S8: summary of MeRIP-seq results (IP vs INPUT for sh-CTL and sh-FTO samples)

Comparisons	IP vs INPUT	Normalized peak enrichment sh-CTL (mean ratio IP/INPUT +/- SE)	Normalized peak enrichment sh-FTO (mean ratio IP/INPUT +/- SE)
Number of peaks found in known genes	3 458	2.98 +/- 0.023	3.03 +/- 0.026
Number of corresponding genes	1 530		
Number of intergenic peaks	217	2.95 +/- 0.13	2.75 +/- 0.19
Number of unknown peaks	832	3.04 +/- 0.05	2.98 +/- 0.05

Supplementary Methods

Cell cycle analysis

Cell pellets of one million of cells were left one ice for 30 min. Then cells were resuspend in 500 µL of propidium iodide staining buffer (0.1 % of Triton X-100, 0.2 mg / mL Propidium Iodide (ThermoFisher Scientific). After one night of incubation at 4 °C in dark, cell cycle was analyzed using MACSQuant flow cytometer.

RNA purification

mRNA was purified from total RNA with 2 rounds of GeneElute mRNA purification kit (Sigma). rRNA was removed using Ribominus kit (Invitrogen) according to the manufacturer's instructions. Small RNA were purified from total using miRVANA kit according to the manufacturer's instructions (Invitrogen)

MeRIP-sequencing

RNA fragmentation and purification

5 µg of mRNA were fragmented (Invitrogen AM8740) by adding 1 µL of 10X fragmentation reagent to 9 µL mRNA and incubated at 95˚C for 1 min. The same samples were then pooled prior to overnight EtOH precipitation (0.1 volume of 3M sodium acetate pH5.2, 2.5 volume of pure EtOH, 1 µL glycogen). The fragmented mRNA were recovered by centrifugation at 14,000rpm 25 min at 4˚C and the pellets were washed once with 70% EtOH and centrifuged again 14,000rpm 10 min at 4 ˚C. The pellets were resuspended in 429 µL DEPC water and 390 µL was used for m^6A-methylated RNA immunoprecipitation and 39 µL for inputs (10%).

RNA immunoprecipitation

390 µL of fragmented mRNA was diluted with 100 µL of 5X IP buffer (250mM Tris pH7.4, 500mM NaCl, 0.25% NP-40) and incubated with 2x(quantity of mRNA) µg of m6A antibody (ab151230) in presence of RNase inhibitors, for 3 hrs at 4˚C. 30 µL of prewashed Surebeads Protein A magnetic beads (BioRad) were added and incubated for 2 hrs at 4˚C. The beads were then washed twice with high-salt IP buffer

(50mM Tris pH7.4, 1M NaCl, 1mM EDTA, 1% NP-40), twice with 1X IP buffer and finally once with high-salt IP buffer.

RNA isolation

200 µL of PK buffer (100mM Tris-HCl pH7.5, 50mM NaCl, 10mM EDTA) and 10 µL of Proteinase K (Invitrogen AM2548) were added to the beads and incubated at 1,100rpm for 20 min at 55˚C. A second elution was performed at 1,100rpm for 10 min at 55˚C and pooled with the first eluates. To isolate RNAs, 1:1 Phenol:Chloroform was added and incubated at 1,100rpm for 5 min at 30˚C. The aqueous phase containing the RNA and the inputs were EtOH precipitated overnight and recovered to an equal final volume of 16 µL. ¼ of the RNA were used for RT-qPCR (see below) and 10 µL were subjected to sequencing.

RT-qPCR

To validate the IP, RT-qPCR of inputs mRNA and immunoprecipitated mRNA was performed. The RNA were reverse transcribed using the VILO Superscript (Invitrogen™ 11755050) according to the manufacturer's instructions. qPCRs were performed using primers for GAPDH, known not to be m6A modified, and for MYC 3'UTR.

Primers for qPCR

GAPDH_Fwd TGGTATCGTGGAAGGACTCA

GAPDH_Rev CCAGTAGAGGCAGGGATGAT

MYC_Fwd (m6A peak in 3'UTR) CATCAGCACAACTACGCAGC

MYC_Rev (m6A peak in 3'UTR) GCTGGTGCATTTTCGGTTGT

Sequencing

Multiplex cDNA Library was produced using NEBNext Small RNA Library Prep Set for Illumina (NE Biolabs[Inc] E7330S), according to manufacturer procedure. Sequencing was performed using NextSeq 500.

Me-RIP Analysis

MeRIP-seq libraries read quality were assessed using FastQC v0.11.5 (Babraham Institute, Cambridge, UK) and adaptors were filtered using cutadapt v2.10 **[1]**. Ribosomal RNAs were discarded using SortMeRNA v2.1b **[2]**. High quality reads were then aligned on the *Homo sapiens* reference genome version GRCh38 using CRAC v2.5 (ATGC, LIRMM, FR). Alignment files were indexed using Samtools v1.9 **[3]**. Detection of m6A peak enrichment and statistical analysis were performed using m6aViewer (University of Leeds, Leeds, UK) **[4]**. Duplicate reads and read not in a proper pairs were discarded from the analysis using options of m6aViewer. Primary risk of probabilities to false discovery fold change was corrected by Benjamini and Hochberg multiple test adjustment. Corrected p-values < at 0.05 % were kept. Gene identifications were performed with biomaRt R package **[5]**. Functional annotations were performed with online program Panther v.15.0 **[6]**. Graphics were realized using R software **[7]** and Pandas python library **[8]**.

To validate MERIP sequencing experiment, we applied the motif inference tool, Homer [homer], on all peaks that are differentially enriched between IP and Input. We sought motifs of length 7, without mismatches; the entire sequence from the peak region is considered (we did not restrict the analysis to shorter windows centered around the peak maximum height). The most significant motif reported by Homer with a P-value of 1e-78 is RWGGACT, which matches the DRACH motif on its last position (GGACT) (**Table S7**). In our condition, the Homer motif is highly specific, as seen on the logo and on its average information content per position (1.82 bits, with a maximum of 2 bits per position); this motif matches 59% (2045) of target sequences. When Homer was run, with one mismatch allowed, for length 6 or 7, the same motif remains significant and appears as number 2 in the Homer list, showing that the motif is robust to variations of Homer parameters.

Transcriptome Analysis.

Total RNA was extracted using TRIzol method. RNA samples were send to Eurofins Genomics /GATC to perform next generation sequencing on Illumina platform.

Polysome fractionation.

This procedure was performed as previously described **[9]**. Briefly, 6 plates (150 cm^2) were seeded with 2 x 10^6 cells. After 72 h, cells were treated with 20 µg / ml emetine for 5 min at 37°C, washed twice with ice-cold PBS, and scraped in ice cold PBS. Cells were centrifuged, resuspended into 1mL of polysome lysis buffer, homogenized by hard shaking with 1,4mm ceramix spheres (Lysing matrix D MPBio) in FastPrep machine (MPBio), centrifuges 10 min at full speed at 4°C. Lysates were loaded on 15-50% sucrose gradient and centrifuged at 35,000 rpm for 2.5 h at 4°C in a SW41 rotor (Beckman Coulter). Polysomes were separated through a live optical density (OD) 254 nm UV spectrometer and collected with an ISCO (Lincoln, NE) density gradient fractionation system. The absorbance at 254 nm was measured continuously as a function of gradient depth.

Translatome Analysis.

mRNA associated with 1 to 3 ribosomes "Light polysomes" and mRNA associated with more than 3 ribosomes "Heavy polysomes" were extracted using TRIzol LS (Invitrogen) according to manufacturer's instructions. Library preparation was performed using TruSeq Stranded mRNA Sample Preparation kit (Illumina) according to the manufacturer's protocol. cDNA libraries were sequenced using the sequencer HiSeq 2500 (Illumina).

Bioinformatic pipeline.

Transcriptome and translatome libraries read quality were assessed using FastQC v0.11.5 (Babraham Institute, Cambridge, UK). Ribosomal RNAs were discarded using SortMeRNA v2.1b **[10]**. High quality reads were then aligned on the *Homo sapiens* reference transcriptome, version GRCh38.cdna, and quantified using pseudocounts with Kallisto v0.45.0 **[11]**. Kallisto quantification parameters were fixed

at 25 for k-mer size for the index, 20 for standard deviation and 100 for bootstraps. Statistical differential analyses were performed on each dataset using Wald test from DESeq2 R package **[12]**. Each count dataset was filtered at 1 count per millions per biological sample after size factors estimation, then dispersion was estimated. Primary risk of probabilities to false discovery fold change was corrected by Benjamini and Hochberg multiple test adjustment. Corrected p-values < at 0.05 % were kept. Volcano plots were realized with ggplot2 R package **[13]**. Gene identifications were performed with biomaRt R package **[14]**. Functional annotations were performed with online gProfileR **[15]** using a g:SCS threshold < at 0.05.

REFERENCES

1. Martin, M. (2010). *Cutadapt removes adapter sequences from high-throughput sequencing reads*. EMBnet.journal.17.1. https://doi.org/10.14806/ej.17.1.200
2. Kopylova, E., et al., SortMeRNA: fast and accurate filtering of ribosomal RNAs in metatranscriptomic data. Bioinformatics, 2012. 28: p. 3211–3217.
3. Li, H., et al. (2009). *The Sequence Alignment/Map format and SAMtools*. Bioinformatics, 15;25(16):2078-9.
4. Antanaviciute, A. (2017). *m6aViewer: software for the detection, analysis, and visualization of N^6-methyladenosine peaks from m^6A-seq/ME-RIP sequencing data*. RNA, 23(10):1493-1501. https://doi.org/10.1261/rna.058206.116.
5. Durinck, S., et al., "Mapping identifiers for the integration of genomic datasets with the R/Bioconductor package biomaRt." Nature Protocols, 2009. 4: p. 1184–1191.
6. Huaiyu, M., et al. (2019). *PANTHER version 14: more genomes, a new PANTHER GO-slim and improvements in enrichment analysis tools*. Nucl. Acids Res. https://doi.org/10.1093/nar/gky1038
7. R core team (2020). R: A language and environment for statistical computing. R Foundation for Statistical Computing. Vienna, Austria. URL https://www.R-project.org/
8. McKinney, W. (2010). *Data Structures for statistical computing in python*. Proc. Of the 9th python in science conf. (SCIPY 2010).
9. David, A., et al., *RNA binding targets aminoacyl-tRNA synthetases to translating ribosomes.* J Biol Chem, 2011. **286**(23): p. 20688-700.
10. Kopylova ,E., et al., SortMeRNA: fast and accurate filtering of ribosomal RNAs in metatranscriptomic data. Bioinformatics, 2012. 28: p. 3211–3217.
11. Bray , N.L., et al., Near-optimal probabilistic RNA-seq quantification. Nature Biotechnology, 2016. 34: p. 525-527.
12. Love, M.I., et al., Moderated estimation of fold change and dispersion for RNA-seq data with DESeq2. Genome Biology, 2014. 15(12): p. 550.
13. Wickham, H. ggplot2: Elegant Graphics for Data Analysis. Springer-Verlag New York, 2016.
14. Durinck, S., et al., "Mapping identifiers for the integration of genomic datasets with the R/Bioconductor package biomaRt." Nature Protocols, 2009. 4: p. 1184–1191.

15. Reimand, J., et al., g:Profiler -- a web server for functional interpretation of gene lists (2016 update). Nucleic Acids Research, 2016. doi:10.1093/nar/gkw199.
