# Supplementary material for: FTO-mediated cytoplasmic m6Am demethylation adjusts stem-like properties in colorectal cancer cell
Source: Nat Commun. 2021 Mar 19;12:1716. doi: 10.1038/s41467-021-21758-4 (PMC7979729; doi:10.1038/s41467-021-21758-4)
Supplement: Supplementary file 4 — Description of Additional Supplementary Files [file 41467_2021_21758_MOESM4_ESM.docx]

SUPPLEMENTARY DATA 1 LEGEND

Provided supplementary data is composed of two sheets by performed analysis i.e. transcriptome, light and heavy fractions. The first sheet (e.g. Transcriptome_norm_counts) contains normalized counts according to DESeq2 normalization process, i.e. counts divided by sample-specific size factors determined by median ratio of gene counts relative to geometric mean per gene. The second sheet (e.g. transcriptome_deseq2) contains the output from differential analysis for all genes with log2FoldChange and p-values. Unormalized counts are provided on the GEO portal GSE165115.
