# Supplementary material for: FTO-mediated cytoplasmic m6Am demethylation adjusts stem-like properties in colorectal cancer cell
Source: Nat Commun. 2021 Mar 19;12:1716. doi: 10.1038/s41467-021-21758-4 (PMC7979729; doi:10.1038/s41467-021-21758-4)
Supplement: Supplementary file 5 — Reporting Summary [file 41467_2021_21758_MOESM5_ESM.pdf]

nature research

Corresponding author(s): Alexandre David
Amandine Bastide
Eric Rivals

Last updated by author(s): Nov 28, 2019

Reporting Summary

Nature Research wishes to improve the reproducibility of the work that we publish. This form provides structure for consistency and transparency in reporting. For further information on Nature Research policies, see Authors & Referees and the Editorial Policy Checklist.

Statistics

For all statistical analyses, confirm that the following items are present in the figure legend, table legend, main text, or Methods section.

n/a	Confirmed	
☐	☒	The exact sample size (*n*) for each experimental group/condition, given as a discrete number and unit of measurement
☐	☒	A statement on whether measurements were taken from distinct samples or whether the same sample was measured repeatedly
☐	☒	The statistical test(s) used AND whether they are one- or two-sided<br>*Only common tests should be described solely by name; describe more complex techniques in the Methods section.*
☒	☐	A description of all covariates tested
☒	☐	A description of any assumptions or corrections, such as tests of normality and adjustment for multiple comparisons
☐	☒	A full description of the statistical parameters including central tendency (e.g. means) or other basic estimates (e.g. regression coefficient) AND variation (e.g. standard deviation) or associated estimates of uncertainty (e.g. confidence intervals)
☐	☒	For null hypothesis testing, the test statistic (e.g. *F*, *t*, *r*) with confidence intervals, effect sizes, degrees of freedom and *P* value noted<br>*Give P values as exact values whenever suitable.*
☒	☐	For Bayesian analysis, information on the choice of priors and Markov chain Monte Carlo settings
☒	☐	For hierarchical and complex designs, identification of the appropriate level for tests and full reporting of outcomes
☒	☐	Estimates of effect sizes (e.g. Cohen's *d*, Pearson's *r*), indicating how they were calculated

Our web collection on statistics for biologists contains articles on many of the points above.

Software and code

Policy information about availability of computer code

Data collection

No software was used. All data came from our experiments.

Data analysis

Bioinformatic pipeline.
Transcriptome and translatome libraries read quality were assessed using FastQC v0.11.5. Ribosomal RNAs were discarded using SortMeRNA v2.1b. High quality reads were then aligned on the Homo sapiens reference transcriptome, version GRCh38.cdna, and quantified using pseudocounts with Kallisto v0.45.0. Kallisto quantification parameters were fixed at 25 for k-mer size for the index, 20 for standard deviation and 100 for bootstraps. Statistical differential analyses were performed on each dataset using Wald test from DESeq2 R package. Each count dataset was filtered at 1 count per millions per biological sample after size factors estimation, then dispersion was estimated. Primary risk of probabilities to false discovery fold change was corrected by Benjamini and Hochberg multiple test adjustment. Corrected p-values < at 0.05 % were kept. Volcano plots were realized with ggplot2 R package. Gene identifications were performed with biomaRt R package. Functional annotations were performed with online gProfileR using a g:SCS threshold < at 0.05.

Me-RIP Analysis.
MeRIP-seq libraries read quality were assessed using FastQC v0.11.5 and adaptors were filtered using cutadapt v2.10. Ribosomal RNAs were discarded using SortMeRNA v2.1b. High quality reads were then aligned on the Homo sapiens reference genome version GRCh38 using CRAC v2.5. Alignment files were indexed using Samtools v1.9. Detection of m6A peak enrichment and statistical analysis were performed using m6aViewer v1.6.0. Duplicate reads and read not in a proper pairs were discarded from the analysis using options of m6aViewer. Primary risk of probabilities to false discovery fold change was corrected by Benjamini and Hochberg multiple test adjustment. Corrected p-values < at 0.05 % were kept. Gene identifications were performed with biomaRt R package. Functional annotations were performed with online program Panther Gene Ontology v.15.0 [6]. Graphics were realized using R v3.5.1 and Pandas python library.

All the scripts used are hosted on a private gitlab depository and could be available from the corresponding author on reasonable request (contact: rivals@lirmm.fr).
Other softwares used in this study:

Zen Blue edition 3.2 (Carl Zeiss)
Flowing Software 2.5.1 (Perttu Terho, University of Turku, Finland)
Image Lab version 3,0 build 11 , Bio-Rad Laboratories)
ImageJ 1.53c (Wayne Rasband, NIH, USA))
LightCycler 480 Software, Version 1.5 .0 (Roche Applied Science)
Prism 8.4.2 (GraphPad Software)
Skyline (64-bit) 19.1.193 (University of Washington)

For manuscripts utilizing custom algorithms or software that are central to the research but not yet described in published literature, software must be made available to editors/reviewers. We strongly encourage code deposition in a community repository (e.g. GitHub). See the Nature Research guidelines for submitting code & software for further information.

Data

Policy information about availability of data

All manuscripts must include a data availability statement. This statement should provide the following information, where applicable:

- Accession codes, unique identifiers, or web links for publicly available datasets
- A list of figures that have associated raw data
- A description of any restrictions on data availability

Our data collections (RNA-seq, POL-seq and MERIP-seq) are freely available on Gene Expression Omnibus NCBI at accession number GSE165115 (https://www.ncbi.nlm.nih.gov/geo/query/acc.cgi?acc=GSE165115).
Reference genome and transcriptome files are available on Ensembl Database at : ftp://ftp.ensembl.org/pub/release-100/fasta/homo_sapiens/
Reference annotation file is available on Ensembl Database at : ftp://ftp.ensembl.org/pub/release-100/gtf/homo_sapiens/

Field-specific reporting

Please select the one below that is the best fit for your research. If you are not sure, read the appropriate sections before making your selection.

☒ Life sciences ☐ Behavioural & social sciences ☐ Ecological, evolutionary & environmental sciences

For a reference copy of the document with all sections, see nature.com/documents/nr-reporting-summary-flat.pdf

Life sciences study design

All studies must disclose on these points even when the disclosure is negative.

Sample size	In vitro experiments was executed without any prior sample size calculation. A sample size calculation was done prior to in vivo experiments (in agreement with ethic rules) and validated by the ethics committee of the Languedoc Roussillon Region.
Data exclusions	No data were excluded from the analysis
Replication	Every experiment was performed using at least three technical replicates. Outlier replicate was removed. The mean of the others replicates was calculated to constitute a biological replicate.
Randomization	For in vitro experiments, samples were randomly allocated into experimental groups.<br>For in vivo experiments, mice were randomized into 4 groups prior to chemo treatment.
Blinding	Blinding was not possible for in vitro experiments since the same investigator was allocating samples into experimental groups and analyzing them.<br>For in vivo experiments, investigators were blinded to group allocation since cells injection and tumor growth analysis were performed by a third party (technician from the facility)

Reporting for specific materials, systems and methods

We require information from authors about some types of materials, experimental systems and methods used in many studies. Here, indicate whether each material, system or method listed is relevant to your study. If you are not sure if a list item applies to your research, read the appropriate section before selecting a response.

Materials & experimental systems

n/a	Involved in the study	
☐	☒	Antibodies
☐	☒	Eukaryotic cell lines
☒	☐	Palaeontology
☐	☒	Animals and other organisms
☐	☒	Human research participants
☐	☒	Clinical data

Methods

n/a	Involved in the study	
☒	☐	ChIP-seq
☐	☒	Flow cytometry
☒	☐	MRI-based neuroimaging

Antibodies

Antibodies used	Anti-METTL3, Abnova cat# H00056339-B01P,Mouse, clone B01P, lot :G2031<br>Anti-METTL14, Sigma cat# HPA038002 Rabbit, polyclonal<br>Anti-WTAP, Santa-Cruz cat# Sc-374280 Mouse, clone D7, lot :C2216<br>Anti-YTHDF1, Abcam cat# ab99080, Rabbit, polyclonal, lot :GR302043-3<br>Anti-YTHDF2, ProteinTech cat# 24744-1-AP, Rabbit, polyclonal lot :22139<br>Anti-ALKBH5, Sigma cat# HPA007196, Rabbit, polyclonal, lot :E107149<br>Anti-FTO, Phosphosolution cat# 597-FTO, mouse, clone: 5-2H10, lot :CH318<br>Anti-FTO, Abcam cat# ab124892, Rabbit, clone EPR6895<br>Anti-PCIF1, ProteinTech cat# 16082-1-AP, Rabbit, polyclonal, lot :7837<br>Anti-ACTIN, Sigma cat# A5441, Mouse, clone AC15, lot :028K4826<br>Anti-CD44, BD biosciences cat# 559942, Mouse, clone G44-26, lot :3281685<br>Anti-CD44v6, Miltenyi Biotec cat#130-111-238, Clone REA706, lot :5160317418<br>Anti-IgG2a, Miltenyi Biotec cat#130-091-836, mouse, clone S43.10, lot :5160317418<br>REA-S control Isotype, Miltenyi Biotec cat#130-104-614, Clone REA293, lot :5170111362<br>Anti-mouse IgG (m-IgGκ BP-HRP), Santa-Cruz cat#Sc-516102<br>Anti-Rabbit IgG HRP-linked, Cell Signaling, cat#7074S<br>Alexa Fluor® 488 Goat Anti-Mouse (IgG), Invitrogen cat#A-11029<br>Anti- m6A , Abcam cat#ab151230, Rabbit polyclonal
Validation	Anti-METTL3 to anti-ACTIN, antibodies were used for western blot applications The band profiles were compared to the manufacturer's one and validated using si-RNA strategy (except for anti-ACTIN).<br>anti-CD44 and anti-CD44v6 antibodies were used for flow cytometry applications. To evaluated the specifity of those antibodies, the intensity of the staining was compared to the ones obtained with their respective isotype control.<br>Other antibodies have been validated in previous studies whose corresponding DOI is indicated above

Eukaryotic cell lines

Policy information about cell lines

Cell line source(s)	HCT-116 - ATCC<br>SW620 - ATCC<br>CRC1 - patient derived cell lines<br>CPP6 - patient derived cell lines<br>CPP14 -patient derived cell lines<br>CPP25 - patient derived cell lines<br>CPP43 - patient derived cell lines<br>CPP30 - patient derived cell lines<br>CPP36 - patient derived cell lines<br>CPP19 - patient derived cell lines<br>CTC44 - patient derived cell lines from liquid biopsies<br>CTC45 - patient derived cell lines from liquid biopsies
Authentication	Cell lines were previously autenticated in the lab but not during this study.
Mycoplasma contamination	CRC1 - HCT116 - SW620- CTC44 and CTC45 were negative for mycoplasma.<br>CPP19, CPP6, CPP30, CPP36, CPP14, CPP25, CPP43 were positive for mycoplasma.
Commonly misidentified lines<br>(See ICLAC register)	No commonly misidentified cell lines were used in the study

Animals and other organisms

Policy information about studies involving animals; ARRIVE guidelines recommended for reporting animal research

Laboratory animals	Hsd:Athymic Nude-Foxn1nu nu/nu, 6 weeks, females<br><br>Housing parameters :<br>Temperature : 22°C<br>Humidity : 55%<br>Day/night cycle : 12h/12h (8AM/8PM during summer / 7AM/7PM during winter)
Wild animals	This study did not involve wild animals.
Field-collected samples	This study did not involve Field-collected samples.
Ethics oversight	These studies were approved by the ethics committee of the Languedoc Roussillon Region and carried out in compliance with the CNRS and INSERM ethical guidelines of animal experimentation (CEEA-LR-12051).

Note that full information on the approval of the study protocol must also be provided in the manuscript.

Human research participants

Policy information about studies involving human research participants

Population characteristics	We used samples collected between 2014 and 2016 in the frame of the Clinical and Biological Database BCBCOLON (registered at ClinicalTrials.gov as NCT03976960). We selected only samples from patients with colorectal cancer who did not receive neoadjuvant treatment and with a formalin-fixed paraffin-embedded sample suitable for TMA building. Overall, 52 samples (6 adenomas, 9 stage I, 10 stage II, 11 stage III, 8 stage IV and 8 metastases) from 45 patients (20 females and 25 males) were used in this study. The patients' median age was 67 years (range: 48–82 years). Each patient was treated according to our institution guidelines.
Recruitment	The recruitment is done in the digestive surgery department of each investigator site in close collaboration with oncologists or gastroenterologists.
Ethics oversight	Protocol has been approved by the french Ethics Comittee : CPP (Comité de Protection des Personnes) Sud Méditerrannée III.

Note that full information on the approval of the study protocol must also be provided in the manuscript.

Clinical data

Policy information about clinical studies

All manuscripts should comply with the ICMJE guidelines for publication of clinical research and a completed CONSORT checklist must be included with all submissions.

Clinical trial registration	NCT03976960<br>The aim of this clinical trial is the developement of a clinical and biological database in colon cancer and colic tumors in order to better understand tumor invasion and metastatic scattering processes. The investigators hope that a better understanding of tumoral invasion process will lead to the discovery of new biomarkers and new drugs. For this reason, a CONSORT diagram is not suitable.
Study protocol	The full protocol is accessible on request at the Clinical and Translational Research Department if the ICM (Montpellier Cancer Institute): Jean-Pierre BLEUSE, M.D 4 67 61 31 02 ext + 33 DRCI-icm105@icm.unicancer.fr
Data collection	The Biological Resources Center NF S 96-900-labelled (DC-2008-695) of the ICM is in charge of the identification and traceability of samples, their storage, their conservation in optimal conditions as well as their transfer if necessary. The inclusion of patients has started in April 2014 and will finish in December 2025. The data and samples collection will continue until 2030.
Outcomes	Primary outcome was defined as the level of nuclear and cytoplasmic FTO expression according to the tumor stage. Level of FTO expression was assessed as described in the manuscript. No other clinical outcome was used in this study.

Flow Cytometry

Plots

Confirm that:

- [x] The axis labels state the marker and fluorochrome used (e.g. CD4-FITC).
- [x] The axis scales are clearly visible. Include numbers along axes only for bottom left plot of group (a 'group' is an analysis of identical markers).
- [x] All plots are contour plots with outliers or pseudocolor plots.
- [x] A numerical value for number of cells or percentage (with statistics) is provided.

Methodology

Sample preparation	Cells cultured in adherence are dissociated using trypsin and then stain using ALDefluor kit (Stem Cell Technology)<br>Cells culture in suspension are dissociated using Accumax and then stained with anti-CD44 or anti-CD44v6 antibodies (according to manufacturer's instructions)
Instrument	MACSQuant Analyer (Miltenyi)
Software	Flowing Software
Cell population abundance	The final population consists of at least 10 000 cells
Gating strategy	Total population was visualized in FSC / SSC graph. The main population was gated.<br>Then, the cell doublets were removed using FSC- H / FSC- A graph.<br>The dead cells was removed from analysis using FSC / V1 (Sytox blue staining) graph.<br>The population negative for sytox blue staining was gated.

The population of alive singulets cells was used for final analysis. The control gates contain up to 1% of the total number of cells and consist on the background of the staining. The same gates were used to analysis the staining in different conditions.

☒ Tick this box to confirm that a figure exemplifying the gating strategy is provided in the Supplementary Information.
